# Benefits of Physiotherapy Interventions in Survivors of Childhood Cancer: A Systematic Review with Meta-Analysis

**DOI:** 10.3390/cancers18050855

**Published:** 2026-03-06

**Authors:** Lucía Ortiz-Comino, Tania María Abril-Mera, Miguel Ángel Fernández-Gualda, Mario Lozano-Lozano, Fahed Herbawi, Carolina Fernández-Lao

**Affiliations:** 1 Biomedical Group (BIO277), Department of Physical Therapy, Health Sciences Faculty, University of Granada, 18071 Granada, Spain; luciaoc@ugr.es (L.O.-C.); mguelangel@correo.ugr.es (M.Á.F.-G.); mlozano@ugr.es (M.L.-L.); carolinafl@ugr.es (C.F.-L.); 2 Instituto de Investigación Biosanitaria, ibs.GRANADA, 18012 Granada, Spain; 3 Sport and Health Research Center (IMUDs), Parque Tecnológico de la Salud, 18007 Granada, Spain; 4 Health Sciencies Faculty, Catholic University of Santiago de Guayaquil, Guayaquil 090615, Ecuador; tania.abril@cu.ucsg.edu.ec; 5 Department of Allied and Applied Medical Sciences, Faculty of Medicine and Health Sciences, An-Najah National University, Nablus P.O. Box 7, Palestine

**Keywords:** childhood cancer, health-related quality of life, physiotherapy, exercise interventions, fatigue

## Abstract

**Background**: Survival rates of pediatric and childhood cancer are about 80% in 5 years, which suggests that side effects may appear a while after oncological treatment and can be associated with other health impairments. Early rehabilitation interventions, such as exercise-based physiotherapy, help reduce side effects and maintain an adequate physical condition, thereby improving daily capacity and health-related quality-of-life (HRQoL). The purpose of this systematic review with meta-analysis is to demonstrate which are the most common strategies performed in child and adolescent survivors of childhood cancer to improve their HRQoL and their physical condition. **Methods**: Two reviewers searched four databases to identify studies that evaluated the effects of physiotherapy and exercise interventions in child and adolescent survivors of childhood cancer. **Results:** Nine studies performing different exercise interventions were included. The most commonly evaluated outcomes were HRQoL, fatigue, and depression. Seven studies were included in the meta-analysis, with no significant results achieved. **Conclusions**: Aerobic interventions are the most common strategies performed in child and adolescent survivors of childhood cancer to improve their HRQoL. Depression and fatigue seem to improve with these interventions, but more research is needed to confirm these results. Our meta-analysis revealed inconsistent results supporting the use of exercise interventions in this population.

## Simple Summary

To maintain an adequate physical condition, exercise-based physiotherapy is recommended in cancer survivors. This systematic review with meta-analysis aims to describe which are the most common strategies used in child and adolescent survivors of childhood cancer. Although our results are too inconsistent to support the use of exercise interventions in this population, outcomes such as quality-of-life, depression and fatigue seem to improve with aerobic interventions in child and adolescent survivors of cancer.

## 1. Introduction

Pediatric and childhood cancer forms 1% of all diagnosed cancers globally: annually, 400,000 children worldwide will be diagnosed with childhood cancer [1,2]. This type of cancer differs from cancers developed in adults [3], as the most common cancers diagnosed in children can be divided into hematologic tumors (leukemia, Hodgkin Lymphoma) or solid tumors (brain tumors, sarcomas, and other specific tumors such as neuroblastomas or retinoblastomas) [4]. Among all types of cancer, leukemia is the most common in children between 0 and 14 years of age, regardless of geographical area [3].

The survival rates of childhood cancer are approximately 80% in 5 years, when families can afford adequate treatment and care [2]. However, in some cases, this high rate of survival suggests that side effects may appear a while after oncological treatment; side effects resulting not only from the treatment, but also from the cancer itself. These side effects may start as a consequence, but can be associated with other health impairments in life [5,6]. Some of the most frequent physical impairments are obesity, due to changes in corporal composition, pulmonary and cardiac dysfunction, fatigue, changes in the musculoskeletal system (e.g., discoordination, reduced range of motion), and pain, all of which lead to psychological impairments such as depression or anxiety [7]. Accordingly, this population has a higher risk of perceiving a reduced health-related quality-of-life (HRQoL) [8].

Based on current evidence, early rehabilitation interventions may help reduce the incidence of these side effects following oncological treatment [9]. It is assumed that exercise-based physical therapy interventions help to maintain an adequate physical condition, improving daily capacity and HRQoL [10,11]. Accordingly, several studies have reported beneficial effects of exercise-based interventions [9,11,12], yet there is not enough evidence to confirm which interventions are most effective for addressing side effects in childhood cancer populations [13]. Moreover, no previous systematic reviews with meta-analyses have focused on HRQoL, which highlights a gap concerning the most effective interventions to improve patient outcomes.

The main objective of this study is to demonstrate which are the most common strategies performed in child and adolescent survivors of childhood cancer to improve their HRQoL. It is also of interest in this review to review if physical condition, as well as other outcomes such as fatigue and depression, are also improved by physical therapy interventions. The objective of this meta-analysis was to investigate which intervention is the most significant to improve HRQoL in child and adolescent survivors of childhood cancer.

## 2. Materials and Methods

### 2.1. Protocol and Registration

Following the Preferred Reporting Items for Systematic Reviews and Meta-Analyses (PRISMA) guidelines [14], a systematic review was conducted to achieve our objectives. All details concerning the protocol undertaken for this systematic review were registered on the International Prospective Registry of Systematic Reviews (PROSPERO) and are allowed to be accessed at www.crd.york.ac.uk/PROSPERO (accessed on 24 May 2023; registration code: CRD42023428022). The question posed for this review, as recommended by the PRISMA guidelines, was “Are physiotherapy interventions in survivors of childhood cancer improving their quality-of-life?”

### 2.2. Eligibility Criteria

The following criteria were established in order to select which studies were included in this review: (1) experimental studies, (2) studies with participants that were survivors of childhood cancer, (3) participants aged between 6 and 18 years old, (4) any physical therapy rehabilitation intervention, (5) full text accessible on online, and (6) studies published in English, French or Spanish. Studies that did not meet these criteria, such as other article types, unpublished data, or studies performed in animals or in vitro, were excluded.

### 2.3. Search and Information Sources

Searches were performed in April 2021 using the Medline (via PubMed search engine), Scopus, Web of Science, and The Cochrane Library databases. The search strategy was based on MeSH terms and keywords related to childhood cancer and physiotherapy strategies to achieve the proposed outcomes, combined with Boolean operators and search techniques tailored to each database (Supplementary Materials). In addition, the reference lists of the retrieved reports were manually searched for additional references.

### 2.4. Study Selection

This systematic review was conducted independently by two authors (LOC and TMAM), who initially screened articles by title and abstract and then by the full text, evaluating them according to the previously determined inclusion and exclusion criteria. This selection was performed by using the software Rayyan (http://rayyan.qcri.org/) [15]. When a disagreement arose between reviewers, an external reviewer (CFL) decided whether the article should be included or excluded. After both screenings were completed, the search was re-executed to verify if there were any additional studies that could be included in this review (latest search conducted on 12 June 2025).

### 2.5. Data Collection Process and Data Items

Data collection was carried out independently by two authors (LOC and TMAM). One of them selected the data, and then, to verify its accuracy, the information was checked by the second author. In case of doubt, a third researcher (CFL) was asked. The collected data items were: (1) first author, (2) year of publication, (3) country, (4) study design, (5) clinical entity responsible for the study, (6) sample size, (7) type of intervention(s), (8) number of follow-ups, (9) details of control or comparison groups, and (10) main findings. Regarding the participants’ characteristics, their sex, mean age, stage of cancer, type of cancer, and oncological treatment history were also recorded. Finally, data concerning the frequency, duration, outcome measures, adverse events, type of intervention (both the intervention and the control groups), and results (in terms of mean change and *p*-values) were collected.

### 2.6. Summary Measures

To be eligible, studies should have evaluated any of the main outcomes of this review. These were classified into three different domains: (1) quality-of-life, (2), physical function, and (3) psychological and emotional aspects. Domains could be measured using any validated subjective or objective tool. When available, changes affecting these domains were observed and recorded from baseline to the last reported follow-up.

### 2.7. Assessment of Risk of Bias

Two independent authors reported the quality of the studies included in this systematic review (FH and LOC) by the use of specific scales depending on the type of study, following the instructions given by the Cochrane Handbook for Systematic Reviews of Intervention [16] and the National Institutes of Health (NIH) [17]. When in doubt, a third reviewer (MLL) was consulted.

Randomized controlled trials (RCTs) were evaluated through the Revised Cochrane risk-of-bias tool for randomized trials (RoB 2) [18]. This tool is widely used to evaluate the quality of RCTs and has been updated in the last year to improve its quality. Biases were assessed in five distinct domains (e.g., randomization process, intended interventions, missing data, measurements, and results).

### 2.8. Meta-Analysis

After extracting all of the selected data, and considering the heterogeneity of the included studies assessed by I^2^ (less than 25%, no heterogeneity; 25–49%, low heterogeneity; 50–74%, moderate heterogeneity; and 75% or greater, high heterogeneity) [19], the possibility of performing a meta-analysis was considered. To be included in the meta-analysis, at least two studies should evaluate the selected outcomes with the same tool; this allowed us to evaluate HRQoL, depression, fatigue, and muscle strength. In order to homogenize the results of each study, the results were divided by subgroups depending on the follow-up evaluations, using the mean and standard deviations or available data to calculate them [20]. A random effects model of the DerSimonian and Laird method, that considers variations within and between studies, was used and forest plots were developed to visualize the study summaries and pooled estimates of each outcome analyzed. Cohen’s D was calculated for each of the original studies and an overall estimator with produced STATA software (StataCorp. 2019; Stata Statistical Software: Release 16; StataCorp LLC, College Station, TX, USA); a two-sided *p* value < 0.05 was considered statistically significant.

## 3. Results

**Figure 1:**
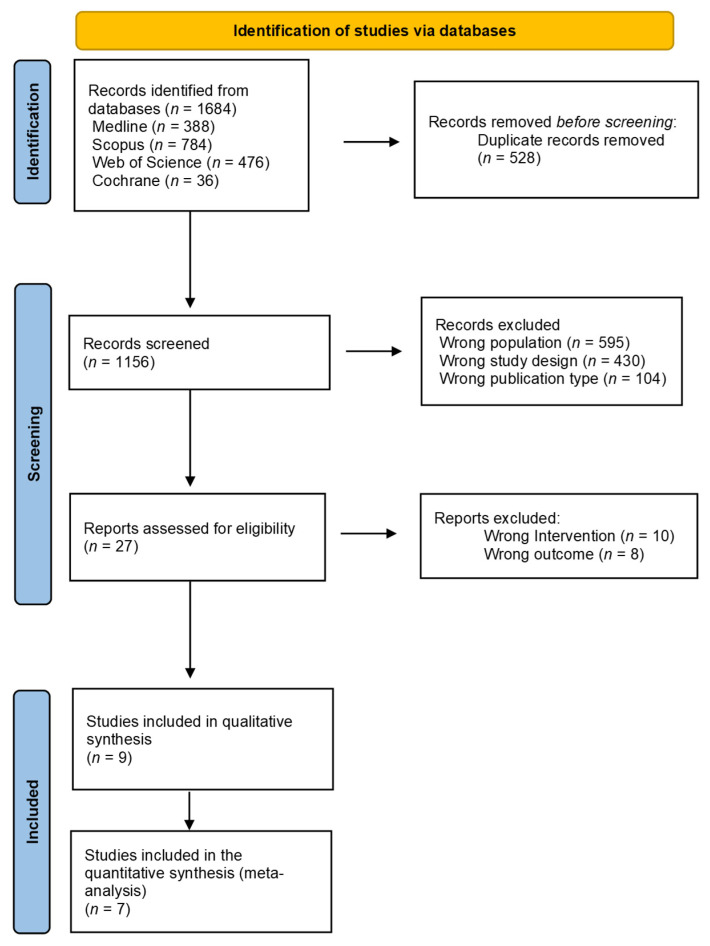
PRISMA flowchart diagram of the search process and selected studies.

Following the literature search, 1684 articles were identified, of which 528 were duplicates and were therefore excluded. A total of 1156 articles were screened by title and abstract, excluding a total of 1129, and resulting in 27 articles that were retrieved for full-text review. Eighteen articles were finally excluded as they were not RCTs or had children undergoing active treatment. Thus, nine articles were included in this systematic review, and seven were included in the meta-analysis. The different stages of the review process (e.g., study identification, inclusion, and exclusion) are shown in the PRISMA flowchart (Figure 1). Interrater agreement in the selection of studies was 70% [21]. After discussion, the reviewers reached a consensus (100%).

### 3.1. Descriptive Synthesis

Of the nine studies included in this review, three were conducted in China [22,23,24]; two in Canada [25,26]; two in the Netherlands [27,28], one in Israel [29] and another one in the USA [30]. A total of 646 subjects participated in the studies included in this review, comprising 337 males and 309 females. The study by Li et al. (2018) [22] had the largest sample size (*n* = 222), and the smallest sample size was found in the study by Dubnov-Raz et al. (*n* = 22) [29]. As indicated on our inclusion criteria, age ranged from a mean of 5.62 [25,26] to a mean of 13.2 [27]. Most of the participants had a diagnosis of a hematologic tumor (*n* = 383) followed by solid tumors (*n* = 239) and other tumors (*n* = 24). Only one study [27] did not report any oncological treatment, but most survivors had received chemotherapy (*n* = 382), surgery (*n* = 151), radiotherapy (*n* = 101), or bone marrow transplant (*n* = 9) (Table 1).

Regarding the physical therapy modalities, three studies were focused only on exercise interventions [25,26,29], three studies combined exercise interventions with psychological interventions [27,28,30], two studies performed an exercise intervention based on adventure training [22,23] and one study combined adventure training with psychological interventions [24]. Table 2 describes in detail all of the specifications regarding the different interventions used.

In terms of duration and frequency, the interventions lasted from 12 weeks to 6 months, with the highest-frequency interventions consisting of four sessions a week [26] and three sessions a week [25,29]. Two studies reported a frequency of two sessions of exercise interventions a week and one session of psychological intervention every two weeks [27,28], and three studies only reported four days of intervention during 6 months [22,23,24]. Only the study by Howell et al. did not report the frequency of their intervention [30]. Table 2 describes the duration and frequency of all included studies.

Quality-of-life was the most evaluated outcome among all the included studies (seven in total) and all of them used the PedsQL questionnaire [22,23,24,27,28,29,30]. Other outcomes such as fatigue, depression, and different outcomes of physical activity were also evaluated with different instruments, as seen in Table 2.

### 3.2. Adverse Events

Only two studies [26,27] indicated that no adverse events occurred during the intervention. All other studies did not mention any information about possible adverse events.

### 3.3. Risk of Bias in the Included RCTs

**Figure 2:**
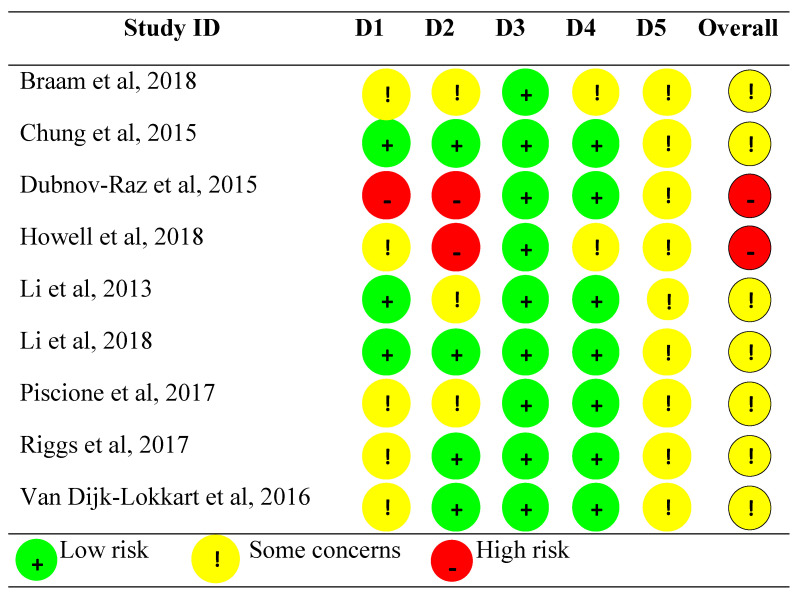
Risk of bias of the included studies [22,23,24,25,26,27,28,29,30]. D1: Randomization process; D2: deviations from the intended interventions; D3: missing outcome data; D4: measurement of the outcome; D5: selection of the reported result.

The great majority of the methodological quality issues (high-risk) were a consequence of deviations from the intended interventions (22%) and the randomization process (11%). In contrast, low-risk percentages were reported for the missing outcome data (100%) and the measurement of the outcome (77%). However, there were many concerns in the selection of the reported results (100%), the randomization process (55%), the deviations from the intended intervention (33%), and some concerns in the measurement of the outcome (22%); thus, none of the studies reached a “low” overall risk of bias. Figure 2 shows an assessment of the risk of bias and methodological quality for each of the included studies.

### 3.4. Qualitative Analysis

The studies of Li et al. and Dijk-Lokkart et al. [22,28], demonstrated an intergroup significant difference in favor of the IG on HRQoL (*p* < 0.05), whereas three studies [24,27,30], showed an intragroup significant difference on this outcome in the intervention group (IG) (*p* < 0.05). In contrast, Chung et al. [23] showed an intragroup significant difference in both the IG and the control group (CG) for HRQoL (*p* < 0.05).

Depression was evaluated in three studies [27,28,29], but only Braam et al. [27] showed an statistically significant intragroup difference (*p* < 0.05) in the CG. Regarding fatigue, Li et al. [22] were the only ones that showed an significant intragroup difference (*p* < 0.05). The results of the other secondary outcomes are shown in Table 2.

### 3.5. Meta-Analysis

#### 3.5.1. Health-Related Quality-of-Life

**Figure 3:**
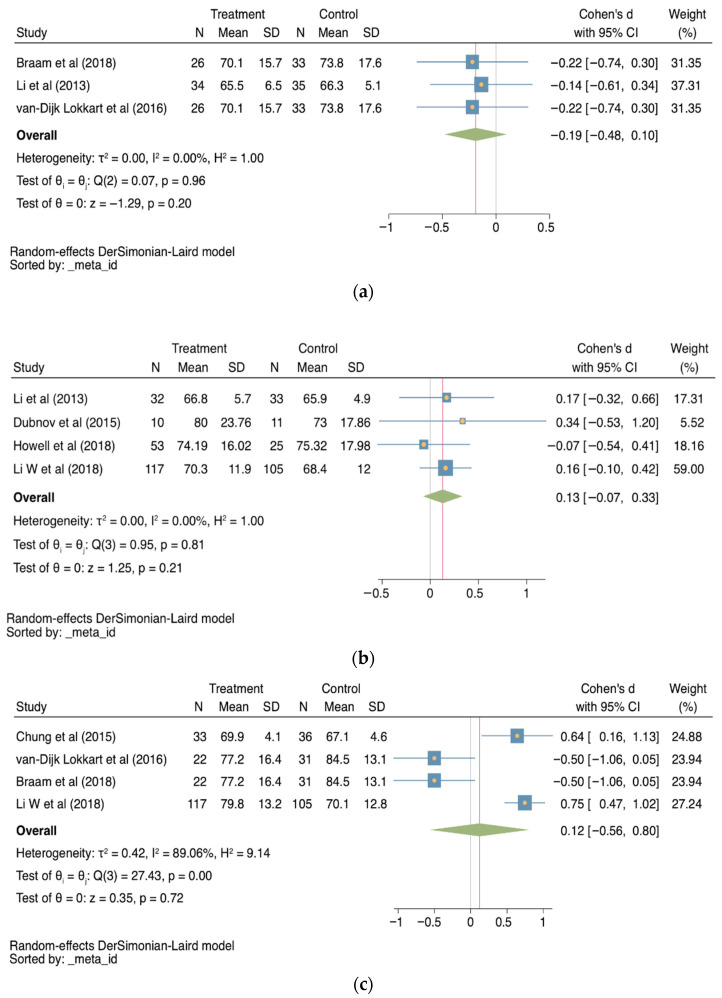
Forest plots for HRQoL (**a**) at three months [24,27,28], (**b**) at 6 months [22,24,29,30], and (**c**) at 12 months [22,24,29,30], comparing treatment to control groups. CI: Confidence interval; N: sample size; SD: standard deviation; τ^2^: between-study variance; I^2^: percentage of variation due to heterogeneity; H^2^: ratio of total variance to within-study variance; Q: Cochran’s Q test; *p*: *p*-value; z: z-score.

**Figure 4:**
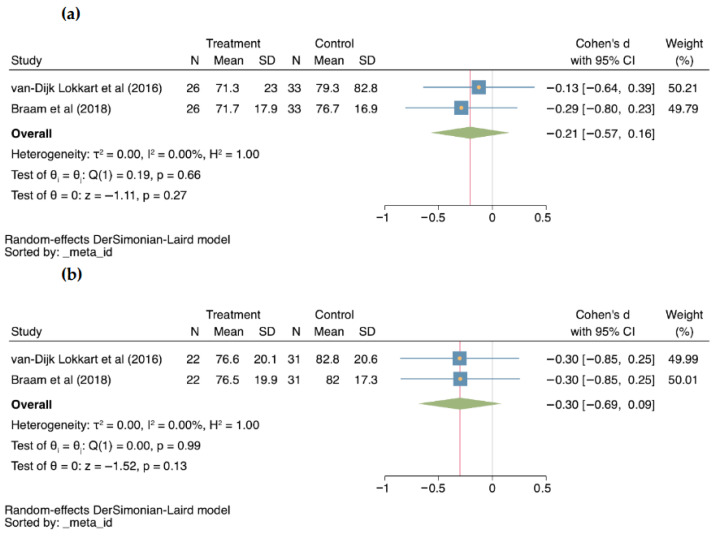
Forest plots for Fatigue; (**a**) at 4 months [27,28], (**b**) at 12 months [27,28], comparing treatment to control groups. CI: Confidence interval; N: Sample size; SD: Standard Deviation; τ^2^, between-study variance; I^2^: percentage of variation due to heterogeneity; H^2^: ratio of total variance to within-study variance; Q: Cochran’s Q test; *p*: *p*-value; z: z-score.

Regarding HRQoL, three studies evaluated this outcome at three months [24,27,28], obtaining a Cohen’s D of −0.19 (95% CI −0.48−0.10; *p* = 0.20, I^2^ = 0%; Figure 3a). Four studies [22,24,29,30] evaluated HRQoL at 6 months (0.13, −0.07–0.33; CI 95%, *p* = 0.21; I^2^ = 0%; Figure 3b) and 4 studies [22,23,27,28] evaluated HRQoL at 12 months (0.12, CI 95% −0.56–0.80; I^2^ = 89.06%, *p* = 0.72; Figure 3c), with no statistically significant results at any evaluation point (*p* > 0.05).

#### 3.5.2. Fatigue

Fatigue was evaluated by two studies at 4 months (−0.21; 95% CI −0.57–0.16; I^2^ = 0%; *p* = 0.27; Figure 4a) [27,28] and by the same two studies at 12 months (0.30 95% CI −0.69–0.09; I^2^ = 0%; *p* = 0.13; Figure 4b) [27,28]. No statistically significant differences were obtained in both evaluations.

#### 3.5.3. Depression

**Figure 5:**
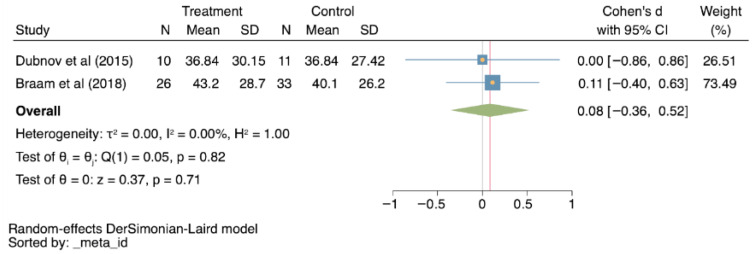
Forest plots for depression, comparing treatment to control groups [27,29]. CI: Confidence interval; N: sample size; SD: standard deviation; τ^2^: between-study variance; I^2^: percentage of variation due to heterogeneity; H^2^: ratio of total variance to within-study variance; Q: Cochran’s Q test; *p*: *p*-value; z: z-score.

Depression was also evaluated by two studies [27,29] (0.08; 95% CI −0.36–0.52; I^2^ = 0% *p* = 0.71; Figure 5), but no significant differences were found between groups for this outcome.

#### 3.5.4. Muscle Strength

**Figure 6:**
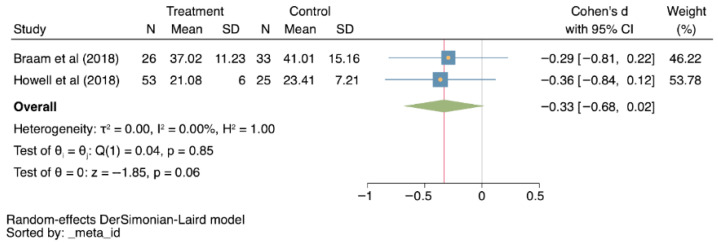
Forest plots for muscle strength, comparing treatment to control groups [27,30]. CI: Confidence interval; N: sample size; SD: standard deviation; τ^2^: between-study variance; I^2^: percentage of variation due to heterogeneity; H^2^: ratio of total variance to within-study variance; Q: Cochran’s Q test; *p*: *p*-value; z: z-score.

Lastly, muscle strength was also evaluated by two studies [27,30] (−0.33; −0.68–0.02; I^2^ = 0%; *p* = 0.06; Figure 6), with no statistically significant differences between the interventions.

## 4. Discussion

This systematic review with meta-analysis was performed to determine which strategies are most commonly performed in child and adolescent survivors of childhood cancer to improve their HRQoL and to establish if other outcomes such as physical condition, fatigue, or depression are also improved by these physical therapy interventions. Based on our results, exercise interventions are the most common strategies employed to improve HRQoL in this population; however, our meta-analysis did not reveal statistically significant differences when comparing this intervention with other therapeutic strategies.

The population of this systematic review was child and adolescent survivors of childhood cancer, mainly affected by hematologic tumors, followed by solid tumors or other neoplasms. Hematologic tumors comprise a broad spectrum of neoplasms and represent the most common type of cancer during childhood [31] followed by solid tumors such as brain and other central nervous system tumors [31]. Many determinants (i.e., cancer variables and their treatment, treatment-related symptoms, and sociodemographic factors) [32] explain the variability of quality-of-life in this population, thus it is of importance to manage these consequences with interventions that focus on their improvement [33].

Regarding HRQoL, it was the most evaluated outcome of this systematic review, but only two studies [22,28] showed an intergroup significant difference in favor of the IG compared to the CG, whereas Braam et al. (2018), Howell et al. (2018), Li et al. (2013), [24,27,30] showed an intragroup significant difference on this outcome for the IG (*p* < 0.05). Finally, Chung et al. [23] showed an intragroup significant difference in both the IG and CG for HRQoL (*p* < 0.05). In concordance with these results, a recent systematic review reported that exercise interventions improve HRQoL in the childhood cancer population, although no meta-analysis was conducted [34]. These effects of exercise interventions on HRQoL are not only stated for the cancer population, but in the general population, as these interventions promote better physical and mental health, as well as psychological well-being [35,36].

Aerobic fitness, physical activity, muscle strength, and other physical condition outcomes were evaluated in all studies. In general, the IGs exhibited significant improvements following the intervention, regardless of the type of intervention; however, in some studies, the CG also showed significant differences after the intervention, even if the children only received their usual care. It may be understandable that, although some side effects such as fatigue may appear with time, in children that are growing up, physical activity abilities may increase over time once they have finished oncological treatment, and their perception of physical activity levels may change even if they do not participate in physical activity interventions [37].

None of the studies that evaluated depression found statistically significant changes for the IG. Although physical activity has been shown to stimulate the release of hormones such as dopamine and serotonin [38,39], there is still a gap in the literature regarding the type, intensity, dosage, and duration, among other items, to allow us to relate physical activity to brain function in the childhood population [39]. Taking into account that children with cancer have more symptoms to address, more research is needed to establish the effects of physical therapy on depression.

Fatigue was reduced after physical activity intervention in only one study [22], based on adventure training. However, in contrast with our results, a recent systematic review with meta-analysis has shown that exercise interventions reduce fatigue on this population [40]. However, they also state that it is of importance that exercise interventions are designed to be game-based, ensuring that children do not feel fatigued while following a physical activity protocol and thus increasing adherence to stimulate motivation [41]. Moreover, these game-based interventions could ease the process of hospital admission [42].

### 4.1. Strengths and Limitations

This systematic review with meta-analysis has some strength points, as it has been written according to PRISMA guidelines. Moreover, a deep assessment risk of bias was performed, and the meta-analysis enhances the results of the qualitative synthesis. Lastly, this research included studies in three different languages, which led to the inclusion of more articles in the inclusion criteria.

Additionally, most studies in this systematic review had small sample sizes, which reduces the statistical power and significance of the results. The heterogeneity in intervention types and variability in outcome measurements further constrain the strength and generalizability of our conclusions. Finally, although not included in the meta-analysis, some studies were included in the systematic review despite differences in methodological quality. These limitations highlight the need for cautious interpretation of the findings and underscore the importance of larger, methodologically rigorous studies to provide more definitive evidence.

### 4.2. Clinical Implications

Based on our results, aerobic interventions during and after oncological treatment are recommended to improve HRQoL in childhood cancer. However, these results are not consistent enough to provide specific indications to clinicians.

### 4.3. Future Research Directions

Future research should determine the optimal type, intensity, and duration of exercise interventions to improve HRQoL, physical fitness, and psychological outcomes in childhood cancer survivors. Conducting larger, multicenter trials with standardized protocols could reduce heterogeneity and allow more precise evaluation in specific patient populations. Furthermore, incorporating engaging, game-based interventions may improve adherence, while further research on fatigue, depression, and long-term effects is warranted.

## 5. Conclusions

Aerobic interventions are the most common strategies performed in children and adolescent survivors of childhood cancer to improve their HRQoL. Depression and fatigue seem to improve with these interventions, but more research is needed to confirm these results. Our meta-analysis revealed inconsistent results supporting exercise interventions applied to children and adolescent survivors of childhood cancer.

## Figures and Tables

**Table 1:** Patients’ characteristics.

Author	Country	Study Design	Sample Size	Gender	Age (Mean, CI, SD)	Cancer Type			Oncological Treatment (*n*, %)
						Hematologic Tumor	Solid Tumor	Other Neoplasms	
Braam et al., 2018 [27]	The Netherlands	RCT	68	37 male 31 female	13.2 (8–18)	45	23	0	Not reported
Chung et al., 2015 [23]	China	RCT—follow-up	69	36 male 33 female	12.6 (2.1 SD)	51	0	18	Chemotherapy (48)
Dubnov-Raz et al., 2015 [29]	Israel	RCT	22	10 male 12 female	10.8 (7.8–13.8)	16	4	2	Chemotherapy (22) Radiotherapy (6) Bone marrow transplant (9)
Howell et al., 2018 [30]	USA	RCT	78	35 male 43 female	12.7 (11–15)	27	47	4	Chemotherapy (63) Radiotherapy (29) Surgery (73)
Li et al., 2013 [24]	China	RCT	63	33 male 30 female	12.7 (2.1 SD)	48	15	0	Chemotherapy (44) Radiotherapy (2) Surgery (5) Mixed (12)
Li et al., 2018 [22]	China	RCT	222	118 male 104 female	12.6 (9–16)	150	72	0	Chemotherapy (157) Radiotherapy (8) Surgery (17) Mixed (40)
Piscione et al., 2017 [25]	Canada	RCT (crossover)	28	16 male 12 female	5.62 (1.92–9.33)	0	28	0	Chemotherapy (24) Radiotherapy (28) Surgery (28)
Riggs et al., 2017 [26]	Canada	RCT (crossover)	28	16 male 12 female	5.62 (1.92–9.33)	0	28	0	Chemotherapy (24) Radiotherapy (28) Surgery (28)
Van Dijk-Lokkart et al., 2018 [28]	The Netherlands	RCT	68	36 male 32 female	12.8 (8–18)	46	22	0	Chemotherapy and/or radiotherapy (68)

CI: Confidence interval; RCT: randomized controlled trial; SD: standard deviation; USA: United States of America.

**Table 2:** Summary of the interventions of the included studies.

Author	Sample Size	Intervention	Frequency	Duration	Comparison	Outcome Measures	Measured Time Points	Adverse Events	Results
Braam et al., 2018 [27]	68	PET (aerobic + weight-bearing exercises) 66–77% HR_peak_ first month, 77–90% HR_peak_ last month + PST (expression of feelings, self-perception, coping skills) *QLIM study*	2 PET per week 1 PST every two weeks	12 weeks	Usual care	**HrQoL** (PedsQL) **Fatigue** (PedsQL—Multidimensional fatigue scale) **Depression** (CDI) **Behavioral problems** (YS-R) **Aerobic fitness** (Godfrey protocol, VO_2 peak_) **Muscle strength** (hand-held dynamometer) **Physical activity** (accelerometer) **Athletic competence** (SPP) **Body composition** (%FM + L1-L4 BMD)	Baseline, week 12, 12-month follow-up	No	**Between groups** Long-term intervention Lower-body muscle strength (IG > CG) *p* < 0.05 All other *p* > 0.05 in both short and long term **Within groups** Increased General HrQoL in both groups (*p* < 0.05) Decrease in depressive symptoms in CG (*p* < 0.05) Increased BMD in both groups (*p* < 0.05) Increased muscle strength in the IG (*p* < 0.05) Increased physical activity in CG (*p* < 0.05)
Chung et al., 2015 [23]	33 IG 36 CG	4-day integrated adventure-based training	4 days	6 months	Standard medical care	**HRQoL** (PedsQL) **Physical activity levels** (PARCY) **Stages of change in physical activity** (PASCQ) **Self-efficacy** (PA-SE)	Baseline (T1), 12 months (T2) and 18 months (T3) after starting the intervention	Not reported	**Between groups** Not reported **Within groups** HRQoL improved in IG from T1 to T2, T1 to T3 and T2 to T3 (*p* < 0.05) Physical activity levels are improved in both groups from T1 to T2 and from T1 to T3 (*p* < 0.05) Self-efficacy improved in IG from T1 to T2 (*p* < 0.05); it also improved in both groups from T1 to T3 (*p* < 0.05)
Dubnov-Raz et al., 2015 [29]	10 IG 12 CG	Warm-up: stationary bikes, treadmills or free running for 15′ Main component: Strength and endurance for 30′ Cool-down period: walking, abdominal crunches, stretching for 15′	3 days a week	6 months	Usual lifestyle habits	**HrQoL** (PedsQL) **Depression** (CDI) **Maximal cardiopulmonary exercise test** (ergometer) **Aerobic fitness** (VO_2peak_) **BC, BMD and BMC** (absorptiometry)	Baseline, 6 months after starting the intervention	Not reported	**Between groups** Not reported **Within groups** BC: Lean body mass improved in IG (*p* < 0.05) Lumbar spine BMD improved in IG (*p* < 0.05) Total BMC improved in IG (*p* < 0.05)
Howell et al., 2018 [30]	53 IG 25 CG	Educational materials Activity monitoring Access to an interactive website to motivate increased physical activity with rewards	Not reported	24 weeks	Educational materials and activity monitoring	**HRQoL** (PedsQL) **Physical activity** (accelerometer) **Fitness** (handgrip dynamometer, sit-ups, push-ups) **General intelligence** (WASI)	Baseline, 24 weeks after starting the intervention	Not reported	**Between groups** No changes between IG and CG (*p* > 0.05) **Within groups** All outcomes improved in IG from baseline to 24-week follow-up (*p* < 0.05)
Li et al., 2013 [24]	34 IG 37 CG	Adventure-based training Health education program (educational talks and workshops)	4 days	6 months	Medical follow-up 4 days of leisure activities	**HRQoL** (PedsQL) **Physical activity levels** (PARCY) **Stages of change in physical activity** (PASCQ) **Self-efficacy** (PA-SE)	Baseline (T1), 3 (T2), 6 (T3), and 9 (T4) months after starting the intervention	Not reported	**Between groups** Improved PASCQ in IG compared to CG (*p* < 0.001) Higher PASCQ in IG compared to CG (*p* < 0.001) Better self-efficacy in IG compared to CG (*p* = 0.04) **Within groups** Better HRQoL, physical activity levels and self-efficacy from T1 to T4 in the IG (*p* < 0.001)
Li et al., 2018 [22]	117 IG 105 CG	Adventure-based training	4 days	6 months	4 days of health talks and leisure activities	**HRQoL** (PedsQL) **Fatigue** (FS-C) **Physical activity levels** (PARCY) **Self-efficacy** (PA-SE)	Baseline (T1), 6 (T2) and 12 (T3) months after starting the intervention	Not reported	**Between groups** Lower fatigue in IG compared to CG at T3 (*p* < 0.01); higher HRQoL, physical activity and self-efficacy in IG compared to CG at T3 (*p* < 0.01) **Within groups** Not reported
Piscione et al., 2017 [25]	14 IG 12 CG	Warm-up (10–15 min) Moderate–vigorous intensity (45–50 min) Cool-down (10 min) Social snack (15 min)	3 sessions of 90 min per week	12 weeks	No training	**Motor function** (BOT-2) **Aerobic fitness** (VO_2peak_) **Fitness** (pro-rated work rate)	Baseline (T1), 12 (T2) and 24 (T3) weeks after starting the intervention	Not reported	**Between groups** Not reported **Within groups** Improved bilateral coordination in IG at T2 and T3 (*p* < 0.05) Improved pro-rated work rate in IG at T2 and T3 (*p* < 0.05)
Riggs et al., 2017 [26]	14 IG 12 CG	Aerobic activities	2 group sessions of 90 per week 2 individual sessions of 30 min per week	12 weeks	No training	**Physical fitness** (6MWT) **Motor function** (BOT-2) **Neuropsychological evaluation** (CANTAB) **Brain structures** (MRI): white matter and hippocampal volume (measured with FA)	Baseline (T1), 12 (T2) and 24 (T3) weeks after starting the intervention	Not reported	**Between groups** Not reported **Within groups** Improved reaction time in the IG (*p* < 0.05) Increased FA in both groups (*p* < 0.05) Increased hippocampal volume in the IG
Van Dijk-Lokkart et al., 2018 [28]	22 IG 31 CG	PET (cardiorespiratory and muscle strength training) + PST (socio-emotional functioning and coping with disease-related effects) *QLIM study*	PET: 2 individual sessions of 45 min per week PST: 1 group session every 2 weeks and 2 parent sessions at the start and the end of the program	12 weeks	Care as usual	**HRQoL** (PedsQL) **Depression** (CDI) **Behavioral problems**: (CBC/YS-R > 11 years old) **Athletic competence** (SPP)	Baseline (T1), 4 (T2) and 12 (T3) months follow-up	Not reported	**Between groups** Less Pain and Hurt in HRQoL in the IG compared to the CG (*p* < 0.05) at T2 and T3 Less Nausea reported in the CG compared to IG at long term (*p* < 0.05) No other changes were seen (*p* > 0.05) **Within groups** Not reported

6MWT: Six-minute walking test; BC: body composition; BMC: bone mineral content; BMD: bone mineral density; BOT-2: Bruininks–Oseretsky test of motor proficiency 2; CANTAB: Cambridge neuropsychological test automated battery; CBC: children behavior checklist; CDI: children depression inventory; CG: control group; FA: fractional anisotropy; FS-C: fatigue scale—child; FM: fat mass; HR_peak_: heart rate peak; HRQoL: health-related quality-of-life; IG: intervention group; MRI: magnetic resonance imaging; PARCY: physical activity rating for children and youth; PASCQ: physical activity stages of change questionnaire; PA-SE: physical activity self-efficacy; PedsQL: pediatric quality-of-life inventory; PET: physical exercise training; PST: psychological training; SPP: self-perception profile; WASI: Wechsler abbreviated scale of intelligence; YS-R: youth self-report.

## Data Availability

The data that support the findings of this study are available from the corresponding author upon reasonable request.

## Supplementary Materials

The following supporting information can be downloaded at https://www.mdpi.com/article/10.3390/cancers18050855/s1, Search strategies tailored to each database; PRISMA 2020 Checklist. Reference [43] is cited in the Supplementary Materials.

## Abbreviations

The following abbreviations are used in this manuscript:

6MWT	Six-Minute Walking Test
BC	Body Composition
BMC	Bone Mineral Content
BMD	Bone Mineral Density
BOT-2	Bruininks–Oseretsky Test of Motor Proficiency 2
CANTAB	Cambridge Neuropsychological Test Automated Battery
CBC	Children Behavior Checklist
CDI	Children Depression Inventory
CG	Control Group
CI	Confidence Interval
FA	Fractional Anisotropy
FS-C	Fatigue Scale—Child
FM	Fat Mass
HR_peak_	Heart Rate peak
HRQoL	Health-Related Quality-of-Life
IG	Intervention Group
MRI	Magnetic Resonance Imaging
NIH	National Institutes of Health
PARCY	Physical Activity Rating for Children and Youth
PASCQ	Physical Activity Stages of Change Questionnaire
PA-SE	Physical Activity Self-Efficacy
PedsQL	Pediatric Quality-of-Life Inventory
PET	Physical Exercise Training
PRISMA	Preferred Reporting Items for Systematic Reviews and Meta-Analyses
PROSPERO	International Prospective Registry of Systematic Reviews
PST	Psychological Training
RCT	Randomized Controlled Trial
ROB-2	Revised Cochrane Risk-Of-Bias Tool For Randomized Trials
SD	Standard Deviation
SPP	Self-Perception Profile
USA	United States of America
WASI	Wechsler Abbreviated Scale of Intelligence
YS-R	Youth Self-Report
