# Supplementary material for: Benefits of Physiotherapy Interventions in Survivors of Childhood Cancer: A Systematic Review with Meta-Analysis
Source: Cancers (Basel). 2026 Mar 6;18(5):855. doi: 10.3390/cancers18050855 (PMC12984369; doi:10.3390/cancers18050855)
Supplement: Supplementary file 1 [file cancers-18-00855-s001.zip › cancers-4153543-supplementary.pdf]

Supplementary Material 1

Search strategies tailored to each database

PUBMED

(((((((((((Child[MeSH Terms])) OR (Adolescent[MeSH Terms])) OR (child-hood[Title/Abstract])))) AND ((((((((((((((((((((neoplasms[MeSH Terms]) OR (carcino-ma[MeSH Terms])) OR (leukemia[MeSH Terms])) OR ("brain neoplasms"[MeSH Terms])) OR ("central nervous system neoplasms"[MeSH Terms])) OR (lymphoma[MeSH Terms])) OR (carcinoma[Title/Abstract])) OR (leukemia[Title/Abstract])) OR ("brain neo-plasm"[Title/Abstract])) OR ("brain neoplasms"[Title/Abstract])) OR ("central nervous system neoplasms"[Title/Abstract])) OR ("central nervous system neo-plasm"[Title/Abstract])) OR (lymphoma[Title/Abstract])) OR (cancer[Title/Abstract])) OR (cancers[Title/Abstract])) OR (tumor[Title/Abstract])) OR (tumors[Title/Abstract])) OR (tumour[Title/Abstract])) OR (tumours[Title/Abstract])))))) AND ((rehabili-tation[MeSH Terms]) OR (acupuncture[MeSH Terms])) OR ("acupuncture therapy"[MeSH Terms])) OR (massage[MeSH Terms])) OR (acupressure[MeSH Terms])) OR ("exercise therapy"[MeSH Terms])) OR ("exercise therapies"[MeSH Terms])) OR (exercise[MeSH Terms])) OR (gymnastic[MeSH Terms])) OR (gymnastics[MeSH Terms])) OR ("physical therapy modalities"[MeSH Terms])) OR ("physical therapy modality"[MeSH Terms])) OR ("activities of daily living"[MeSH Terms])) OR (yoga[MeSH Terms])) OR (qigong[MeSH Terms])) OR ("gong, qi"[MeSH Terms])) OR ("tai ji"[MeSH Terms])) OR ("tai ji quan"[MeSH Terms])) OR ("quan, tai ji"[MeSH Terms])) OR ("breathing exercises"[MeSH Terms])) OR (hydrotherapy[MeSH Terms])) OR (hydrotherapies[MeSH Terms])) OR ("plyometric ex-ercise"[MeSH Terms])) OR ("exercises, plyometric"[MeSH Terms])) OR ("exercise movement techniques"[MeSH Terms])) OR ("exercise movement technics"[MeSH Terms])) OR ("resistance training"[MeSH Terms])) OR ("exercise, muscle stretching"[MeSH Terms])) OR ("muscle stretching exercises"[MeSH Terms])) OR ("physical therapy spe-cialty"[MeSH Terms])) OR ("myofunctional therapy"[MeSH Terms])) OR ("myofunctional therapies"[MeSH Terms])) OR ("electric stimulation therapy"[MeSH Terms])) OR ("musculoskeletal manipulations"[MeSH Terms])) OR ("Manual lymphatic drain-age"[Title/Abstract])) OR ("endurance training"[Title/Abstract])) OR (physiothera-py[Title/Abstract])) OR ("Physical

therapy"[Title/Abstract])) OR ("Physical therapy mo-dalities"[Title/Abstract])) OR (Physiotherapies[Title/Abstract])) OR (exer-cise[Title/Abstract])) OR (exercises[Title/Abstract])) OR ("dry needling"[Title/Abstract])) OR (acupressure[Title/Abstract])) OR (shiatsu[Title/Abstract])) OR (shiatzu[Title/Abstract])) OR ("myofunctional therapy"[Title/Abstract])) OR ("soft tissue therapy"[Title/Abstract])) OR ("high intensity interval training"[Title/Abstract])) OR ("motion therapy"[Title/Abstract])) OR ("myofascial therapy"[Title/Abstract])) OR ("my-ofascial induction"[Title/Abstract])) OR ("massage therapies"[Title/Abstract])) OR (mas-sage[Title/Abstract])) OR ("musculoskeletal manipulations"[Title/Abstract])) OR ("ma-nipulation therapy"[Title/Abstract])) OR ("manipulative therapy"[Title/Abstract])) OR (bodywork[Title/Abstract])) OR ("craniosacral massage"[Title/Abstract])) OR ("manual therapy"[Title/Abstract])) OR ("strength training"[Title/Abstract])) OR ("exercise train-ing"[Title/Abstract])) OR ("aerobic exercises"[Title/Abstract])) OR ("physical activi-ty"[Title/Abstract])) OR ("physical activities"[Title/Abstract])) OR ("physical exer-cise"[Title/Abstract])) OR ("physical exercises"[Title/Abstract])) OR (aero-bic[Title/Abstract])) OR (gymnastics[Title/Abstract])) OR ("acute exercise"[Title/Abstract])) OR ("isometric exercise"[Title/Abstract])) OR ("plyometric exercise"[Title/Abstract])) OR ("resistance training"[Title/Abstract])) OR ("exercise movement tech-niques"[Title/Abstract])) OR ("pilates-based exercises"[Title/Abstract])) OR ("pilates training"[Title/Abstract])) OR (qigong[Title/Abstract])) OR ("tai ji"[Title/Abstract])) OR ("tai chi"[Title/Abstract])) OR (yoga[Title/Abstract])) OR ("breathing exercis-es"[Title/Abstract])) OR ("passive stretching"[Title/Abstract])) OR ("active stretch-ing"[Title/Abstract])) OR ("relaxed stretching"[Title/Abstract])) OR (hydrothera-py[Title/Abstract])) OR ("exercise therapy"[Title/Abstract])) OR ("dance thera-py"[Title/Abstract])) OR ("proprioceptive neuromuscular facilitation"[Title/Abstract])) OR ("therapeutic electrical stimulation"[Title/Abstract])) OR ("therapeutic electri-cal"[Title/Abstract])) OR ("therapeutic electric stimulation"[Title/Abstract])) OR (electro-therapy[Title/Abstract])) OR (electroacupuncture[Title/Abstract])) OR ("spinal cord stim-ulation"[Title/Abstract])) OR (TENS[Title/Abstract])) OR ("transcutaneous electrical stim-ulation"[Title/Abstract])) OR ("transdermal electrostimulation"[Title/Abstract])) OR (electroanalgesia[Title/Abstract])) OR ("radiofrequency treatment"[Title/Abstract])) OR ("low-level light therapy"[Title/Abstract])) OR (photobiomodulation thera-py[Title/Abstract])) OR (photobiomodulation[Title/Abstract])) OR ("laser thera-

py"[Title/Abstract])) OR ("laser therapies"[Title/Abstract])) OR (phototherapy[Title/Abstract])) OR (laser[Title/Abstract])) OR ("transcutaneous electric nerve stimu-lation"[MeSH Terms])) OR ("transcutaneous electrical nerve stimulation"[MeSH Terms])) OR ("electroacupuncture"[MeSH Terms])) OR ("pulsed radiofrequency treatment"[MeSH Terms])) OR ("pulsed radiofrequency treatments"[MeSH Terms])) OR ("pulsed radio frequency treatment"[MeSH Terms]))))))) AND ((((((((((((((((quality of life[MeSH Terms]) OR (human physical conditioning[MeSH Terms])) OR (fatigue[MeSH Terms])) OR (quality of life[Title/Abstract])) OR ("health-related quality of life"[Title/Abstract])) OR ("health related quality of life"[Title/Abstract])) OR (HRQOL[Title/Abstract])) OR (life quality[Title/Abstract])) OR (QOL[Title/Abstract])) OR (cardiorespiratory fit-ness[Title/Abstract])) OR (fatigue[Title/Abstract])) OR (cancer-related fa-tigue[Title/Abstract])) OR (cancer related fatigue[Title/Abstract])) OR (physical condi-tion[Title/Abstract])) OR (human physical condition[Title/Abstract])))) AND ((("Clinical Trials as Topic"[Mesh])) OR ("Controlled Clinical Trials as Topic"[Mesh])) OR ("Intention to Treat Analysis")) OR ("Clinical Trial" [Publi-cation Type])) OR ("Randomized Controlled Trial" [Publication Type])) OR ("Equivalence Trial" [Publication Type])) OR ("Pragmatic Clinical Trial" [Publication Type])) OR ("Controlled Clinical Trial" [Publication Type])) OR ("clinical trials")) OR ("randomized controlled trial")) OR ("randomised controlled trial")) OR ("randomized controlled trials")) OR ("randomised controlled trials")) OR ("controlled clinical trial")) OR ("Controlled Clinical Trials")) OR ("random allocation")) OR ("Double-Blind Method")) OR ("Double Blind Method")) OR ("Single-Blind Method")) OR ("Cross-Over Studies")) OR ("double blind procedure")) OR ("single blind procedure")) OR ("crossover procedure")) OR ("clinical trial")) OR ("controlled study")) OR ("randomization")) OR ("rct")) OR ("single blind")) OR ("double blind")) OR ("Control Groups"[Mesh])) OR ("Control Group")) OR ("Control Groups")) OR ("Placebos"[Mesh])) OR ("Sham Treatment")) OR ("Placebo Ef-fect"[Mesh])) OR ("Placebo Effects")) OR ("Placebo Effect"))))))

SCOPUS

(((TITLE-ABS-KEY ("child" OR "adolescent" OR "childhood")) AND (TI-TLE-ABS-KEY ("carcinoma" OR "leukemia" OR "brain neoplasms" OR "brain neoplasm" OR "central nervous system neoplasms" OR "central nervous system neoplasm" OR

"lymphoma" OR "cancer" OR "cancers" OR "tumor" OR "tu-mour" OR "tumours" OR "tumors"))) AND (TITLE-ABS-KEY ("rehabilitation" OR "acupuncture" OR "acupuncture therapy" OR "massage" OR "acupressure" OR "exercise therapy" OR "exercise therapies" OR "exercise" OR "gymnastic" OR "gymnastics" OR "Physical therapy modalities" OR "physical therapy modal-ity" OR "Activities of daily living" OR "yoga" OR "qigong" OR "gong, qi" OR "tai ji" OR "tai ji quan" OR "quan, tai ji" OR "breathing exercises" OR "hydro-therapy" OR "hydrotherapies" OR "plyometric exercise" OR "plyometric exer-cises" OR "exercise movement techniques" OR "exercise movement technics" OR "resistance training" OR "exercise muscle stretching" OR "muscle stretching exer-cises" OR "physical therapy specialty" OR "myofunctional therapy" OR "myo-functional therapies" OR "electric stimulation therapy" OR "musculoskeletal ma-nipulations" OR "manual lymphatic drainage" OR "endurance training" OR "physiotherapy" OR "physical therapy" OR "physical therapy modalities" OR "physiotherapies" OR "exercises" OR "dry needling" OR "shiatsu" OR "shiatzu" OR "myofunctional therapy" OR "soft tissue therapy" OR "high intensity interval training" OR "motion therapy" OR "myofascial therapy" OR "myo-fascial induction" OR "massage therapies" OR "musculoskeletal manipulations" OR "manipulation therapy" OR "manipulative therapy" OR "bodywork" OR "craniosacral massage" OR "manual therapy" OR "strength training" OR "exer-cise training" OR "aerobic exercises" OR "physical activity" OR "Physical activi-ties" OR "physical exercise" OR "physical exercises" OR "aerobic" OR "acute exercise" OR "isometric exercise" OR "resistance training" OR "exercise move-ment techniques" OR "pilates-based exercises" OR "pilates training" OR "tai chi" OR "passive stretching" OR "active stretching" OR "relaxed stretching" OR "dance therapy" OR "proprioceptive neuromuscular facilitation" OR "therapeutic electrical stimulation" OR "therapeutic electrical" OR "therapeutic electric stimula-tion" OR "electrotherapy" OR "electroacupuncture" OR "spinal cord stimulation" OR "TENS" OR "transcutaneous electrical stimulation" OR "transdermal stimula-tion" OR "electroanalgesia" OR "radiofrequency treatment" OR "low-level light therapy" OR "photobiomodulation therapy" OR "photobiomodulation" OR "laser therapy" OR "laser therapies" OR "phototherapy" OR "laser" OR "transcuta-neous electrical nerve stimulation" OR "pulsed radiofrequency treatment" OR "pulsed radiofrequency treatments")) AND (TITLE-ABS-KEY ("quality of life" OR "human physical conditioning" OR "fatigue" OR "health-related quality of life" OR "HRQOL" OR "health related quality of life"

OR "life quality" OR "QOL" OR "cardiorespiratory fitness" OR "cancer-related fatigue" OR "cancer related fatigue" OR "physical condition" OR "human physical condition") AND (TITLE-ABS-KEY ("Clinical Trials as Topic" OR "Controlled Clinical Trials as Topic" OR "Intention to Treat Analysis" OR "Clinical Trial" OR "Randomized Controlled Trial" OR "Equivalence Trial" OR "Pragmatic Clinical Trial" OR "Controlled Clinical Trial" OR "clinical trials" OR "randomized controlled trial" OR "randomised controlled trial" OR "randomized controlled trials" OR "randomised controlled trials" OR "Controlled Clinical Trials" OR "random allocation" OR "Double-Blind Method" OR "Double Blind Method" OR "Single-Blind Method" OR "Cross-Over Studies" OR "double blind procedure" OR "single blind procedure" OR "crossover proce-dure" OR "clinical trial" OR "controlled study" OR "randomization" OR "rct" OR "single blind" OR "double blind" OR "Control Groups" OR "Control Group" OR "Control Groups" OR "Placebos" OR "Sham Treatment" OR "Placebo Effect" OR "Placebo Effects" OR "Placebo Effect"))))

WEB OF SCIENCE

(((TS=("child" OR "adolescent" OR "childhood")) AND (TS=("carcinoma" OR "leu-kemia" OR "brain neoplasms" OR "brain neoplasm" OR "central nervous system neo-plasms" OR "central nervous system neoplasm" OR "lymphoma" OR "cancer" OR "cancers" OR "tumor" OR "tumour" OR "tumours" OR "tumors"))) AND (TS=("reha-bilitation" OR "acupuncture" OR "acupuncture therapy" OR "massage" OR "acupressure" OR "exercise therapy" OR "exercise therapies" OR "exercise" OR "gymnastic" OR "gymnastics" OR "Physical therapy modalities" OR "physical therapy modality" OR "Activities of daily living" OR "yoga" OR "qigong" OR "gong, qi" OR "tai ji" OR "tai ji quan" OR "quan, tai ji" OR "breathing exercises" OR "hydrotherapy" OR "hydrothera-pies" OR "plyometric exercise" OR "plyometric exercises" OR "exercise movement techniques" OR "exercise movement technics" OR "resistance training" OR "exercise muscle stretching" OR "muscle stretching exercises" OR "physical therapy specialty" OR "myofunctional therapy" OR "myofunctional therapies" OR "electric stimulation therapy" OR "musculoskeletal manipulations" OR "manual lymphatic drainage" OR "endurance training" OR "physiotherapy" OR "physical therapy" OR "physical therapy modalities" OR "physiotherapies" OR "exercises" OR "dry needling" OR "shiatsu" OR "shiatzu" OR "myofunctional therapy" OR "soft tissue therapy" OR "high intensity

interval training" OR "motion therapy" OR "myofascial therapy" OR "myofascial induction" OR "massage therapies" OR "musculoskeletal manipulations" OR "manipulation therapy" OR "ma-nipulative therapy" OR "bodywork" OR "craniosacral massage" OR "manual therapy" OR "strength training" OR "exercise training" OR "aerobic exercises" OR "physical ac-tivity" OR "Physical activities" OR "physical exercise" OR "physical exercises" OR "aerobic" OR "acute exercise" OR "isometric exercise" OR "resistance training" OR "ex-ercise movement techniques" OR "pilates-based exercises" OR "pilates training" OR "tai chi" OR "passive stretching" OR "active stretching" OR "relaxed stretching" OR "dance therapy" OR "proprioceptive neuromuscular facilitation" OR "therapeutic electrical stimulation" OR "therapeutic electrical" OR "therapeutic electric stimulation" OR "elec-trotherapy" OR "electroacupuncture" OR "spinal cord stimulation" OR "TENS" OR "transcutaneous electrical stimulation" OR "transdermal stimulation" OR "electroanal-gesia" OR "radiofrequency treatment" OR "low-level light therapy" OR "photobiomod-ulation therapy" OR "photobiomodulation" OR "laser therapy" OR "laser therapies" OR "phototherapy" OR "laser" OR "transcutaneous electrical nerve stimulation" OR "pulsed radiofrequency treatment" OR "pulsed radiofrequency treatments")) AND (TS=("quality of life" OR "human physical conditioning" OR "fatigue" OR "health-related quality of life" OR "HRQOL" OR "health related quality of life" OR "life quality" OR "QOL" OR "car-diorespiratory fitness" OR "cancer-related fatigue" OR "cancer related fatigue" OR "physical condition" OR "human physical condition") AND (TS=("Clinical Trials as Topic" OR "Controlled Clinical Trials as Topic" OR "Intention to Treat Analysis" OR "Clinical Trial" OR "Randomized Controlled Trial" OR "Equivalence Trial" OR "Pragmatic Clinical Trial" OR "Controlled Clinical Trial" OR "clinical trials" OR "randomized controlled trial" OR "randomised controlled trial" OR "randomized controlled trials" OR "randomised controlled trials" OR "Controlled Clinical Trials" OR "random allocation" OR "Dou-ble-Blind Method" OR "Double Blind Method" OR "Single-Blind Method" OR "Cross-Over Studies" OR "double blind procedure" OR "single blind procedure" OR "crossover procedure" OR "clinical trial" OR "controlled study" OR "randomization" OR "rct" OR "single blind" OR "double blind" OR "Control Groups" OR "Control Group" OR "Control Groups" OR "Placebos" OR "Sham Treatment" OR "Placebo Effect" OR "Placebo Effects" OR "Placebo Effect"))))

COCHRANE LIBRARY

"child" OR "adolescent" OR "Childhood"

AND

"carcinoma" OR "leukemia" OR "brain neoplasms" OR "brain neoplasm" OR "central nervous system neoplasms" OR "central nervous system neoplasm" OR "lymphoma" OR "cancer" OR "cancers" OR "tumor" OR "tumour" OR "tu-mours" OR "tumors"

AND

"rehabilitation" OR "acupuncture" OR "acupuncture therapy" OR "massage" OR "acupressure" OR "exercise therapy" OR "exercise therapies" OR "exercise" OR "gymnastic" OR "gymnastics" OR "Physical therapy modalities" OR "physical therapy modality" OR "Activities of daily living" OR "yoga" OR "qigong" OR "gong, qi" OR "tai ji" OR "tai ji quan" OR "quan, tai ji" OR "breathing exercises" OR "hydrotherapy" OR "hydrotherapies" OR "plyometric exercise" OR "plyometric exercises" OR "exercise movement techniques" OR "exercise movement technics" OR "resistance training" OR "exercise muscle stretching" OR "muscle stretching exercises" OR "physical therapy specialty" OR "myofunctional therapy" OR "myofunctional therapies" OR "electric stimulation therapy" OR "musculo-skeletal manipulations" OR "manual lymphatic drainage" OR "endurance training" OR "physiotherapy" OR "physical therapy" OR "physical therapy modalities" OR "physiotherapies" OR "exercises" OR "dry needling" OR "shiatsu" OR "shiatzu" OR "myofunctional therapy" OR "soft tissue therapy" OR "high inten-sity interval training" OR "motion therapy" OR "myofascial therapy" OR "myo-fascial induction" OR "massage therapies" OR "musculoskeletal manipulations" OR "manipulation therapy" OR "manipulative therapy" OR "bodywork" OR "craniosacral massage" OR "manual therapy" OR "strength training" OR "exer-cise training" OR "aerobic exercises" OR "physical activity" OR "Physical activi-ties" OR "physical exercise" OR "physical exercises" OR "aerobic" OR "acute exercise" OR "isometric exercise" OR "resistance training" OR "exercise move-ment techniques" OR "pilates-based exercises" OR "pilates training" OR "tai chi" OR "passive stretching" OR "active stretching" OR "relaxed stretching" OR "dance therapy" OR "proprioceptive neuromuscular facilitation" OR "therapeutic electrical stimulation" OR "therapeutic electrical" OR "therapeutic electric stimula-tion" OR "electrotherapy" OR "electroacupuncture" OR "spinal cord stimulation" OR "TENS" OR "transcutaneous electrical stimulation" OR "transdermal stimula-tion" OR "electroanalgesia" OR "radiofrequency treatment" OR "low-level light therapy" OR "photobiomodulation therapy" OR "photobiomodulation"

OR "laser therapy" OR "laser therapies" OR "phototherapy" OR "laser" OR "transcuta-neous electrical nerve stimulation" OR "pulsed radiofrequency treatment" OR "pulsed radiofrequency treatments"AND

AND

"quality of life" OR "human physical conditioning" OR "fatigue" OR "health-related quality of life" OR "HRQOL" OR "health related quality of life" OR "life quality" OR "QOL" OR "cardiorespiratory fitness" OR "cancer-related fa-tigue" OR "cancer related fatigue" OR "physical condition" OR "human physical condition"

PRISMA 2020 Checklist

Section and Topic	Item #	Checklist item	Location where item is reported
**TITLE**			
Title	1	Identify the report as a systematic review.	1
**ABSTRACT**			
Abstract	2	See the PRISMA 2020 for Abstracts checklist.	1
**INTRODUCTION**			
Rationale	3	Describe the rationale for the review in the context of existing knowledge.	2
Objectives	4	Provide an explicit statement of the objective(s) or question(s) the review addresses.	2
**METHODS**			
Eligibility criteria	5	Specify the inclusion and exclusion criteria for the review and how studies were grouped for the syntheses.	3
Information sources	6	Specify all databases, registers, websites, organisations, reference lists and other sources searched or consulted to identify studies. Specify the date when each source was last searched or consulted.	3
Search strategy	7	Present the full search strategies for all databases, registers and websites, including any filters and limits used.	3, 25-29
Selection process	8	Specify the methods used to decide whether a study met the inclusion criteria of the review, including how many reviewers screened each record and each report retrieved, whether they worked independently, and if applicable, details of automation tools used in the process.	3
Data collection process	9	Specify the methods used to collect data from reports, including how many reviewers collected data from each report, whether they worked independently, any processes for obtaining or confirming data from study investigators, and if applicable, details of automation tools used in the process.	3
Data items	10a	List and define all outcomes for which data were sought. Specify whether all results that were compatible with each outcome domain in each study were sought (e.g. for all measures, time points, analyses), and if not, the methods used to decide which results to collect.	3
	10b	List and define all other variables for which data were sought (e.g. participant and intervention characteristics, funding sources). Describe any assumptions made about any missing or unclear information.	3
Study risk of bias assessment	11	Specify the methods used to assess risk of bias in the included studies, including details of the tool(s) used, how many reviewers assessed each study and whether they worked independently, and if applicable, details of automation tools used in the process.	4
Effect measures	12	Specify for each outcome the effect measure(s) (e.g. risk ratio, mean difference) used in the synthesis or presentation of results.	n/a
Synthesis methods	13a	Describe the processes used to decide which studies were eligible for each synthesis (e.g. tabulating the study intervention characteristics and comparing against the planned groups for each synthesis (item #5)).	n/a
	13b	Describe any methods required to prepare the data for presentation or synthesis, such as handling of missing summary statistics, or data conversions.	3, 4
	13c	Describe any methods used to tabulate or visually display results of individual studies and syntheses.	4
	13d	Describe any methods used to synthesize results and provide a rationale for the choice(s). If meta-analysis was performed, describe the model(s), method(s) to identify the presence and extent of statistical heterogeneity, and software package(s) used.	4
	13e	Describe any methods used to explore possible causes of heterogeneity among study results (e.g. subgroup analysis, meta-regression).	4
	13f	Describe any sensitivity analyses conducted to assess robustness of the synthesized results.	4
Reporting bias assessment	14	Describe any methods used to assess risk of bias due to missing results in a synthesis (arising from reporting biases).	4
Certainty assessment	15	Describe any methods used to assess certainty (or confidence) in the body of evidence for an outcome.	4

PRISMA 2020 Checklist

Section and Topic	Item #	Checklist item	Location where item is reported
**RESULTS**			
Study selection	16a	Describe the results of the search and selection process, from the number of records identified in the search to the number of studies included in the review, ideally using a flow diagram.	4, 5
	16b	Cite studies that might appear to meet the inclusion criteria, but which were excluded, and explain why they were excluded.	5
Study characteristics	17	Cite each included study and present its characteristics.	5-17
Risk of bias in studies	18	Present assessments of risk of bias for each included study.	9, 19
Results of individual studies	19	For all outcomes, present, for each study: (a) summary statistics for each group (where appropriate) and (b) an effect estimate and its precision (e.g. confidence/credible interval), ideally using structured tables or plots.	
Results of syntheses	20a	For each synthesis, briefly summarise the characteristics and risk of bias among contributing studies.	9, 19
	20b	Present results of all statistical syntheses conducted. If meta-analysis was done, present for each the summary estimate and its precision (e.g. confidence/credible interval) and measures of statistical heterogeneity. If comparing groups, describe the direction of the effect.	9, 19-22
	20c	Present results of all investigations of possible causes of heterogeneity among study results.	19-22
	20d	Present results of all sensitivity analyses conducted to assess the robustness of the synthesized results.	19-22
Reporting biases	21	Present assessments of risk of bias due to missing results (arising from reporting biases) for each synthesis assessed.	19-22
Certainty of evidence	22	Present assessments of certainty (or confidence) in the body of evidence for each outcome assessed.	n/a
**DISCUSSION**			
Discussion	23a	Provide a general interpretation of the results in the context of other evidence.	22, 23
	23b	Discuss any limitations of the evidence included in the review.	23
	23c	Discuss any limitations of the review processes used.	23
	23d	Discuss implications of the results for practice, policy, and future research.	23
**OTHER INFORMATION**			
Registration and protocol	24a	Provide registration information for the review, including register name and registration number, or state that the review was not registered.	2, 3
	24b	Indicate where the review protocol can be accessed, or state that a protocol was not prepared.	2, 3
	24c	Describe and explain any amendments to information provided at registration or in the protocol.	n/a
Support	25	Describe sources of financial or non-financial support for the review, and the role of the funders or sponsors in the review.	24
Competing interests	26	Declare any competing interests of review authors.	24
Availability of data, code and other materials	27	Report which of the following are publicly available and where they can be found: template data collection forms; data extracted from included studies; data used for all analyses; analytic code; any other materials used in the review.	24

From: Page MJ, McKenzie JE, Bossuyt PM, Boutron I, Hoffmann TC, Mulrow CD, et al. The PRISMA 2020 statement: an updated guideline for reporting systematic reviews. BMJ 2021;372:n71. doi: 10.1136/bmj.n71. This work is licensed under CC BY 4.0. To view a copy of this license, visit https://creativecommons.org/licenses/by/4.0/
